# Increased Cardiovascular Risk in Psoriatic Arthritis: Results From a Case-Control Monocentric Study

**DOI:** 10.3389/fmed.2022.785719

**Published:** 2022-05-19

**Authors:** Yannick Degboé, Richard Koch, Laurent Zabraniecki, Bénédicte Jamard, Guillaume Couture, Jean Bernard Ruidavets, Jean Ferrieres, Adeline Ruyssen-Witrand, Arnaud Constantin

**Affiliations:** ^1^Department of Rheumatology, Toulouse University Hospital and University Toulouse III, Toulouse, France; ^2^Department of Epidemiology, INSERM UMR 1027, Toulouse University Hospital, Toulouse, France; ^3^Department of Cardiology, Toulouse University Hospital and University Toulouse III, Toulouse, France

**Keywords:** psoriatic arthritis, cardiovascular risk, cardiovascular events, dyslipidaemia, statins

## Abstract

**Background:**

Psoriatic arthritis (PsA) is associated with increased cardiovascular morbidity and mortality. The aims of our real-life study were to compare the prevalence of cardiovascular risk factors (CVRFs) and cardiovascular events (CVEs) among patients with PsA with a control population, to evaluate the impact of correcting factors in equations that assess cardiovascular risk (CVR) in PsA, and to determine the percentage of patients who reach the LDLc target as indicated by the European guidelines.

**Methods:**

In this observational cross-sectional monocentric case-control study, we used a standardized procedure to systematically assess patients with PsA aged 25–85 years who met the Classification for Psoriatic Arthritis (CASPAR) criteria. Controls were extracted from the MOnitoring NAtionaL du rISque Artériel (MONALISA) study. We compared the prevalence of CVRFs, CVEs, the CVR, and the percentage of patients reaching recommended LDLc target in both populations. The CVR was first assessed using SCORE and QRISK2 equations. Then, the SCORE equation was corrected by applying a 1.5 multiplication factor, as recommended by EULAR for rheumatoid arthritis (SCORE-PsA), and the QRISK2 was corrected using the “rheumatoid arthritis” item (QRISK2-PsA).

**Results:**

A total of 207 PsA and 414 controls were included. CVRFs and CVEs were more frequent in the PsA group. After controlling for age and gender, atherothrombotic disease was increased in the PsA population (SCORE *p* = 0.002, QRISK2 *p* = 0.001). Using the SCORE-PsA increased the percentage of patients with a high or very high CVR from 39.3 to 45.3% in the PsA group. Similarly, using the QRISK2-PsA increased the percentage of patients with a CVR ≥ 10% from 44.9 to 53.2%. The percentages of patients with PsA with high LDLc in the high and very high CVR groups were not significantly different from controls, despite a trend in favor of patients with PsA. Of the 83 PsA with a QRISK2 ≥ 10%, only 22.9% were treated with statin vs. 35.8% of the 134 controls. The QRISK2-PsA score did not alter these results.

**Conclusion:**

In real-life, patients with PsA have a higher prevalence of CVRFs, as well as a higher prevalence of CVEs compared to the general population. The CVR is higher in the PsA population than in the controls either using the SCORE and QRISK2 equations or using the corrected SCORE- PsA and QRISK2-PsA equations.

## Introduction

Psoriatic arthritis (PsA) is a chronic inflammatory rheumatic disease that affects 0.19% of the European population (1). Since 2016, the European Cardiology Association guidelines have included auto-immune inflammatory diseases as an inherent cardiovascular risk factor (CVRF) (2). As is the case with rheumatoid arthritis (RA) (3, 4), many studies have shown an increase in cardiovascular mortality and morbidity (4–6) and more traditional CVRFs in patients with PsA (5–10). Therefore, control of CVRFs is essential. This is exemplified by the decrease in cardiovascular events (CVEs) in RA and patients with PsA treated with statins (11, 12).

The aims of this study were (i) to compare the prevalence of CVRFs and cardiovascular events (CVEs) in patients with PsA and in matched controls from the general population; (ii) to compare the cardiovascular risk (CVR) in both populations with SCORE and QRISK2 equations with or without taking into account the additional risk attributable to PsA; (iii) to compare the proportion of individuals in both populations who reach the recommended low density lipoprotein cholesterol (LDLc) level according to the SCORE equation, and the proportion of individuals treated with statins according to the National Institute for Health and Care Excellence (NICE) recommendations.

## Materials and Methods

### Patients and Controls

Adults with PsA from our Rheumatology Centre (Toulouse University Hospital) who meet the CASPAR criteria were consecutively recruited in this observational cross-sectional case–control study from March 2016 to January 2017 (13).

The control population was from the French MONALISA (14) study, the main objective of which was to estimate the prevalence of CVRFs among adults who were 35–74 years old from 3 French regions. The pairing process included subjects living in the region of our Rheumatology center (Occitanie, France). Controls were paired with cases at a 2:1 ratio, considering gender and age (±2 years).

### Ethics

Participants in this study gave their written informed consent. The study was approved by the local Toulouse University Hospital ethics committee (n°07-0316).

### Data Collection

Patients were assessed according to a standardized procedure including a questionnaire, a physical examination by the patient’s usual rheumatologist, and biological tests. All collected data were computerized and anonymized for analysis.

The questionnaire collected data on (i) PsA characteristics: CASPAR criteria, ACPA (anti-citrullinated peptides antibodies) positivity, medication, and (ii) CVRFs: familial first-degree myocardial infarction or sudden death before the age of 55 in men and 65 in women, diabetes with its treatment and duration, smoking with cessation date and consumption, hypertension (HT) and its treatment, history of CVE with its treatment, and dyslipidaemia and its treatment.

Physical examination included blood pressure measurement after 5 min of rest, twice, with a 5-min interval, sitting or lying down, with an automatic device. We recorded the highest value. We also recorded size, weight, body mass index (BMI), and waist size.

A biological assessment was performed by the patient’s usual laboratory and included fasting blood sugar, C reactive protein (CRP), erythrocyte sedimentation rate (ESR), serum creatinine, and glomerular filtration rate, total cholesterol, HDL cholesterol, and triglycerides. LDLc was calculated with the Friedewald formula.

In the control population from the French MONALISA (14) study, each event reported by the subject during the interview was checked using the population register of ischemic heart disease, which has been active in the region since 1984.

### Definitions

Hypertension was defined as a history of HT, or use of antihypertensive treatment, or blood pressure ≥ 140/90 mmHg during our standardized examination. Diabetes was defined as a history of type 1 or type 2 diabetes mellitus, or the use of antidiabetic treatment. Dyslipidaemia was defined as a history of dyslipidaemia, or use of lipid-lowering treatment, or abnormal lipid levels. Metabolic syndrome was defined according to the National Cholesterol Education Program (NCEP) Adult Treatment Panel III (ATP III) criteria (15). CVEs included myocardial infarction, stroke, and obliterating arteriopathy of the lower limbs (OALL).

### Evaluation of the Cardiovascular Risk

The CVR was first assessed using SCORE and QRISK2 equations (16). Given the absence of any published equation to assess CVR with the QRISK2, we used the online calculator available at https://www.qrisk.org/2016/. Calculation was performed twice for each case and control.

Then, the SCORE equation was corrected by applying a 1.5 multiplication factor as recommended by the European League Against Rheumatism (EULAR) for rheumatoid arthritis patients (SCORE-PsA) and the QRISK2 equation was corrected using the “rheumatoid arthritis” item (QRISK2-PsA) (17). In addition, we performed a specific evaluation of the cardiovascular risk according to the SCORE-PsA equation, in the subset of patients aged 40–65 years, the SCORE equation being validated in this age stratum.

### Statistical Analysis

Univariate analysis described data with mean, extreme values standard deviation (SD) for quantitative parameters, and frequency for categorial data. Homoscedasticity and normality were tested and showed that logarithmic transformation was necessary for the following covariates to reach normality and to stabilize variances: triglycerides concentration, systolic blood pressure, blood glucose level, and CRP. The bivariate analysis considered the 2:1 pairing, each matched pair was considered as an extreme stratified sample, each stratum corresponded to the two subjects of the same pair.

A conditional logistic regression was used to test differences in categorial covariates between cases and controls. Considering each pair as independent, we used mixed linear models to compare quantitative covariates between cases and controls, each pair being considered as random. The calculated scores were represented according to decile distribution and cumulated frequencies. The significance threshold was *p* < 0.05. The analysis was performed with the software SAS version 9.4 (SAS Institute Inc., Cary, NC, United States).

## Results

### Subject Characteristics and Prevalence of Cardiovascular Risk Factors

The main characteristics of the 207 cases and 414 controls are shown in Table 1. The mean age and the gender ratio in both populations were not statistically different.

**TABLE 1:** Characteristics of patients with psoriatic arthritis (PsA) and controls.

	PsA (*n* = 207)	Controls (*n* = 414)	*P*-value
Age, years, mean (SD)	54.7 (11.4)	54.8 (10.7)	NS
Women, n (%)	100 (48.3)	200 (48.3)	NS
Disease duration, years, mean (SD)	11.9 (8.5)	NA	
BMI, kg/m^2^, mean (SD)	26.6 (4.9)	25.2 (4)	0.001
Systolic blood pressure, mmHg, mean (SD)	134 (15)	129 (22)	0.001
Diastolic blood pressure, mmHg, mean (SD)	79 (11)	80 (12)	0.27
Triglycerides, g/L, mean (SD)	1.24 (0.72)	1.06 (0.63)	0.001
LDLc, g/L, mean (SD)	1.26 (0.38)	1.43 (0.35)	0.001
HDLc, g/L, mean (SD)	0.54 (0.14)	0.59 (0.14)	0.001
Total cholesterol, g//, mean (SD)	2.04 (0.43)	2.23 (0.38)	0.001
Blood sugar, g/L, mean (SD)	1.00 (0.28)	0.99 (0.17)	0.65
CRP, mg/L, mean (SD)	7.0 (14.4)	1.8 (1.9)	0.001
Serum creatinine, μmol/l, mean (SD)	73.4 (17.7)	86.5 (14.9)	0.001
Creatinine clearance MDRD, ml/min/m^2^, mean (SD)	91.9 (18.9)	88.3 (17.7)	0.03
HT, n (%)	71 (34.4)	109 (26.3)	0.03
HT treatment, n (%)	64 (30.9)	73 (17.6)	0.001
Diabetes, n (%)	25 (12.1)	32 (7.7)	0.09
Diabetes treatment, n (%)	24 (11.6)	24 (5.8)	0.02
Dyslipidaemia, n (%)	52 (25.1)	174 (42.0)	0.001
Dyslipidaemia treatment, n (%)	30 (14,4)	93 (22.5)	0.14
Smoking			0.04
Never, n (%)	87 (42.0)	199 (48.1)	
Current, n (%)	50 (24.2)	66 (15.9)	
Past, n (%)	70 (33.8)	149 (36.0)	
Metabolic syndrome, n (%)	59 (28.4)	48 (11.6)	0.001
Family history of CVE, n (%)	29 (14.0)	65 (15.7)	0.57
NSAID, n (%)	87 (42.2)	26 (6.3)	0.001
Steroids, n (%)	20 (9.7)	7 (1.7)	0.001
csDMARD, n (%)	112 (54.1)	0 (0)	
bDMARD, n (%)	137 (66.2)	0 (0)	
No DMARD, n (%)	9 (4.3)	382 (92.3)	
Antiplatelet agent, n (%)	17 (8.2)	24 (5.8)	0.23
Anticoagulant, n (%)	7 (3.4)	2 (0.5)	0.02
β-blocker, n (%)	27 (13.0)	35 (8.5)	0.07
ACEi/ARB, n (%)	39 (18.8)	45 (10.9)	0.005

*PsA patients and controls were paired according to age and gender. PsA, psoriatic arthritis; NA, not applicable; NS, non-significant BMI, body mass index; LDLc, low density lipoprotein cholesterol; HDLc, high density lipoprotein cholesterol; CRP, C reactive protein; MDRD, modification of diet in renal disease; HT, hypertension; NSAIDs, non-steroidal antiinflammatory drugs; csDMARD, conventional synthetic Disease Modifying Antirheumatic Drug; bDMARD, biological DMARD; ACEi, angiotensin-converting enzyme inhibitor; ARB, angiotensin II receptors blocker; SD, standard deviation.*

Compared to controls, patients with PsA had a higher CVRFs (BMI, prevalence of HT, triglycerides, CRP, prevalence of smoking, and prevalence of metabolic syndrome). We observed a non-significant trend for a higher prevalence of diabetes (12.1 vs. 7.7%, *p* = 0.09).

In addition, compared to controls, patients with PsA had lower LDLc (g/L; 1.26 ± 0.38 vs. 1.43 ± 0.35, *p* < 0.001), HDLc (g/l; 0.54 ± 0.14 vs. 0.59 ± 0.14, *p* < 0.001), total cholesterol (g/l; 2.04 ± 0.43 vs. 2.23 ± 0.38, *p* < 0.001), serum creatinine (μmol/L; 73.4 ± 17.7 vs. 86.5 ± 14.9, p < 0.001), and prevalence of dyslipidaemia (25.1 vs. 42%, *p* = 0.001).

### Prevalence of Cardiovascular Events

The prevalence of myocardial infarction, stroke, and OALL was numerically higher, without statistically significant difference, in patients with PsA (Table 2), with, respectively 5.8% vs. 2.9% (*p* = 0.08); 2.4% vs. 1% (*p* = 0.18), and 1.5% vs. 0.5% (*p* = 0.23). Overall, CVE was significantly more frequent in the PsA group (8.7% vs. 4.1%, *p* = 0.03).

**TABLE 2:** Prevalence of cardiovascular events in patients with PsA and controls.

	PsA (*n* = 207)	Controls (*n* = 414)	*P*-value
MI, n (%)	12 (5.8)	12 (2.9)	0.08
Stroke, n (%)	5 (2.4)	4 (1.0)	0.18
OALL, n (%)	3 (1.5)	2 (0.5)	0.23
CVE, n (%)	18 (8.7)	17 (4.1)	0.03

*MI, myocardial infarction; OALL, obliterating arteriopathy of the lower limbs; CVE, cardiovascular events.*

### Cardiovascular Risk Assessment With the SCORE and SCORE-PsA Equations

The CVR was estimated for the entire PsA (*n* = 201) and control population (*n* = 402) using the SCORE and the corrected SCORE-PsA equation applying the 1.5 factor recommended by EULAR for RA patients (Table 3 and Supplementary Figure 1) (17). We used a similar approach for the 145 patients with PsA and 286 controls aged 40–65 years (Table 3 and Supplementary Figure 2). The analysis revealed an additional 10-year risk of global cardiovascular mortality in patients with PsA compared to controls, both in the entire population and in patients 40–65 years old, with both the SCORE-PsA and SCORE equations.

**TABLE 3:** Cardiovascular risk according to the SCORE and SCORE-PsA equations.

Risk	PsA (*n* = 201),%	Controls (*n* = 402),%	*P*-value	PsA 40–65 (*n* = 145),%	Controls 40–65 (*n* = 286),%	*P*-value
SCORE			0.002			0.002
Low (<1%)	24.4	30.7		21.4	31.5	
Intermediate (1–4%)	36.3	39.0		43.4	46.5	
High (5–9%)	13.9	16.6		14.5	12.6	
Very high (≥10%)	25.4	13.7		20.7	9.4	
SCORE-PsA			0.001			0.001
Low (<1%)	24.4	30.7		21.4	31.5	
Intermediate (1–4%)	30.4	39		37.5	46.5	
High (5–9%)	17.9	16.6		20	12.6	
Very high (≥10%)	27.4	13.7		20.7	9.4	

### Assessment of the Cardiovascular Risk With the QRISK2 and QRISK2-PsA Equations

The CVR was estimated for the entire PsA (*n* = 186) and control population (*n* = 397) using the QRISK2 and the corrected QRISK2-PsA equation applying the “Rheumatoid arthritis” item (Table 4 and Supplementary Figures 3, 4) (17).

**TABLE 4:** Cardiovascular risk according to QRISK2 and QRISK2-PsA equations.

Risk	PsA (*n* = 186),%	Controls (*n* = 372),%	*P*-value
QRISK2			0.001
<5%	35.1	42.8	
5–9%	20	23.4	
10–19%	26	22.9	
≥20%	18.9	10.8	
QRISK2-PsA			0.001
<5%	29	42.8	
5–9%	17.7	23.4	
10–19%	24.7	22.9	
≥20%	28.5	10.8	

The QRISK2 equation highlighted an additional risk of death and CVE in patients with PsA compared to controls (*p* < 0.001). PsA, being considered a CVRF similar to RA, the proportion of patients with PsA with a CVR ≥ 10% (QRISK2-PsA) increases from 44.9 to 53.2% (Table 4). We observed a global shift in the distribution toward ≥ 10% categories. Overall, using QRISK2-PsA equation predicted a significantly higher risk, with a median score of 8.7 with QRISK2 and 11.3 with QRISK2-PsA (*p* = 0.0248).

### Patients Who Reach the Therapeutic Target According to the SCORE

We then estimated the achievement of the LDLc therapeutic target across the population of cases (*n* = 201) and controls (*n* = 410) or restricted to the population of cases (*n* = 145) and controls (*n* = 286) aged 40–65 years, based on the level of cardiovascular risk derived from the SCORE and SCORE-PsA equations (Table 5). Whether in patients with PsA or matched controls, only a small proportion of individuals achieved the LDLc therapeutic target, particularly for high and very high cardiovascular risk levels according to SCORE and SCORE-PsA equations.

**TABLE 5:** Percentage of patients with PsA at LDLc target according to EU recommendations with SCORE equations.

Estimated risk	PsA (*n* = 201)	Controls (*n* = 410),%	*P*-value	PsA 40–65 (*n* = 145),%	Controls 40–65 (*n* = 286),%	*P*-value
**SCORE**						
<5%	122	286		94	223	
LDLc < 1.15 g/L (%)	50 (41.0)	63 (22)	0.002	39 (41.5)	36 (16.1)	0.001
5–9%	28	68		21	36	
LDLc < 1 g/L (%)	1 (3.6)	3 (4.4)	NS	0 (0)	2 (5.6)	NS
≥10%	51	56		30	27	
LDLc < 0.7 g/L (%)	7 (13.7)	2 (3.6)	NS	5 (16.7)	1 (3.7)	NS
**SCORE-PsA**						
<5%	110	286		86	223	
LDLc < 1.15 g/L (%)	46 (41.8)	63 (22)	0.002	36 (41.9)	36 (16.1)	0.001
5–9%	36	68		29	36	
LDLc < 1 g/L (%)	3 (8.3)	3 (4.4)	NS	2 (6.9)	2 (5.6)	NS
≥10%	55	56		30	27	
LDLc < 0.7 g/L (%)	7 (12.7)	2 (3.6)	NS	5 (16.7)	1 (1.7)	NS

### Patients Who Reach the Therapeutic Target According to QRISK2

The proportion of cases and controls treated with statin according to the NICE recommendations was estimated for the total population of cases (*n* = 186) and controls (*n* = 397) based on the level of cardiovascular risk derived from the QRISK2 and QRISK2-PsA equations (Table 6). In both patients with PsA and controls, we observed a small proportion of individuals with ≥ 10% QRISK2 treated with statins (NICE recommendations): 19/83 (22.9%) in the PsA population vs. 48/134 (35.8%) in controls. The QRISK2-PsA score did not change these findings.

**TABLE 6:** Proportion of statin-treated cases and controls by cardiovascular risk level from the QRISK2 equation as per NICE recommendations.

	PsA (*n* = 186)	Controls (*n* = 397),%	*P*-value
**QRISK2**			
<10%	103	263	
With Statin (%)	5 (4.9)	33 (12.6)	0.05
≥10%	83	134	
With Statin (%)	19 (22.9)	48 (35.8)	NS
**QRISK2-PsA**			
<10%	87	263	
With Statin (%)	2 (2.3)	33 (12.6)	0.008
≥10%	99	134	
With Statin (%)	22 (22.2)	48 (35.8)	NS

## Discussion

This monocentric observational case–control study shows an increase in the prevalence of the main cardiovascular risk factors and an increase in the prevalence of cardiovascular events in patients with PsA compared to controls. The use of the SCORE and QRISK2 risk formulas, with or without RA additional cardiovascular risk adjustment factor (SCORE-PsA and QRISK2-PsA), show an increase in the cardiovascular risk level of patients with PsA as compared to controls. In both patients with PsA and controls, this study found very low proportions of individuals who meet the LDLc objective or who were treated with statins in accordance with the recommendations.

Our study found high triglycerides and significantly lower LDLc, HDLc, and total cholesterol compared to controls. A recent review of 6 studies comparing PsA lipid levels to controls showed the same dyslipidaemia profile as in our study (18). It is recognized that under inflammatory conditions, the production of proinflammatory cytokines alters lipid profile by decreasing HDLc and LDLc (19, 20). The precise mechanism is not understood. With the use of anti-TNF or anti-IL6 agents, the lipid profile of treated patients changes with an increase in total cholesterol, HDLc, and LDLc fractions, and possibly triglycerides (21). Despite this change in lipid profile with bDMARDs, there does not appear to be an additional cardiovascular risk (20, 22, 23).

This work also highlights an increased prevalence of hypertension in patients with PsA compared to controls. HT in PsA and RA is widely described in the literature with a prevalence ratio ranging from 1.3 to 1.9 depending on the study (5, 6, 24, 25). The increase in the prevalence of HT in PsA can be partly explained by a significantly higher BMI in rheumatism, as well as the existence of chronic systemic inflammation (7). In addition, DMARDs can also be a source of hypertension, e.g., NSAIDs and leflunomide (26).

Our data confirm the high prevalence of metabolic syndrome in PsA (27, 28). Since metabolic syndrome is a combination of cardiovascular risk factors, it is a marker of cardiovascular risk. Therefore, acting on this syndrome is important since it could influence the effectiveness of treatment. In fact, metabolic syndrome could be associated with a lower probability of obtaining low PsA activity with antiTNF agents (29).

A total of 5.8% of our PsA population had a history of coronary disease, 2.4% of ischemic stroke, and 1.5% of an arterial disease of the lower limbs, with a numerically higher, although not statistically significant, proportion in PsA compared with controls. On the other hand, the combination of these events reveals a significant additional risk of cardiovascular events in patients with PsA of 8.7% vs. 4.1% in controls (*p* = 0.03). Two other studies found a higher proportion of coronary events, ischemic strokes, and MACE in PsA compared to controls (4, 6), with significantly different results for only one of them (4).

Using the SCORE, our study found an additional risk of cardiovascular mortality in 10 years in patients with PsA as compared to controls. The corrected SCORE results (SCORE-PsA) highlight the additional cardiovascular risk in the PsA population. Two articles did not find this additional risk of cardiovascular mortality in PsA with the SCORE (6, 30). In the study by Gulati et al., the lack of additional risk is explained by the similarity in risk factors between cases and controls. In the study by Rosales et al., the number of RA and controls was low (80 individuals in each group). Another study compared the cardiovascular SCORE levels in Ankylosing spondylitis (SA) and patients with PsA vs. patients with RA. It showed that cardiovascular mortality was significantly higher in RA than in SA, but found no difference between RA and PsA after age and gender adjustments (31). On the other hand, when the SCORE equation of RA was corrected by a factor of 1.5 according to the EULAR recommendations (17), SA and PsA had a significantly lower cardiovascular mortality risk than RA.

Although easy to use and reliable, the SCORE has limitations. First of all, it estimates a fatal cardiovascular risk and not an overall risk. Moreover, the age interval for the table is limited to 40–65 years, although in practice, it can be used outside these limits (2). In addition, the SCORE was created by combining patient cohorts from 12 European countries (16). The cohort was composed of patients recruited between 1967 and 1991 and is therefore outdated. Finally, it does not consider chronic inflammatory diseases as an independent cardiovascular risk factor.

With the QRISK2, our study revealed an additional risk of mortality and cardiovascular events in 10 years in our PsA population. We also applied the “rheumatoid arthritis” item in the QRISK2 calculator for our PsA population (QRISK2-PsA). This increased the proportion of patients with a cardiovascular risk ≥ 10%, eligible for statin treatment according to the NICE recommendations, from 44.9 to 53.2%. This proportion was higher than the real-life prescription in our center (22.9%). Algorithms to estimate CVR usually underestimate CVR in PsA. Published data on the adaptation of CVR equations are limited and need to be optimized for use in PsA (32–34). Since PsA has a previously established CV morbidity–mortality, it should be included in the QRISK2 algorithm as an independent risk factor, in the same manner as RA.

A small proportion of high-risk and very high-risk cases and controls were the goal for LDLc. However, the interpretation of these results should be conservative given the small number of individuals per group. To our knowledge, no study has attempted to assess the LDLc objectives in “real life” in inflammatory rheumatism. In common practice, in the general population, approximately 40% of patients meet the LDLc target (35, 36). Rollefstad et al. organized a therapeutic intervention program according to the CV SCORE level of patients followed for inflammatory rheumatism (RA, SA, and PsA) (37). This study showed that, with an adapted intervention program, 92.1% of RA patients, 90% of SA, and 82.9% of patients with PsA reached the target LDLc after 3 consultations.

The limitations of our study are the cross-sectional design, the small number of patients included related to the short duration of the patient inclusion period, as well as the monocentric design. Moreover, data collection using a questionnaire, although led by an experienced rheumatologist, could be a source of information bias. Finally, we did not stratify the results according to PsA or rheumatologic treatment activity levels which may have a direct impact on cardiovascular risk. The strengths of our study include (i) the matching of 1 case for 2 controls from the Occitanie region, (ii) the use of 2 equations to assess cardiovascular risk levels, and (iii) the application of corrective factors to the SCORE and QRISK2 equations to consider PsA as an inherent cardiovascular risk factor (similar to RA).

## Conclusion

Our study shows an increase in the prevalence of traditional CVRFs, as well as a higher prevalence of CVEs in PsA. The CVR is higher in the PsA population than in the controls using either the SCORE and QRISK2 equations or the corrected SCORE- PsA and QRISK2-PsA equations. Finally, very few cases and controls at high or very high CVR reach the LDLc target and are treated with statins, which highlights the need for treatment optimization.

## Data Availability Statement

The authors confirm that the data supporting the findings of this study are available within the article and/or Supplementary Material.

## Ethics Statement

The studies involving human participants were reviewed and approved by the Toulouse University Hospital Ethics Committee (n°07-0316). The patients/participants provided their written informed consent to participate in this study.

## Author Contributions

RK, JR, JF, and AC designed the study. RK collected the data. YD, RK, LZ, BJ, GC, JR, AR-W, JF, and AC analyzed and interpreted the data. All authors wrote and revised the manuscript and contributed to the article and approved the submitted version.

## Conflict of Interest

The authors declare that the research was conducted in the absence of any commercial or financial relationships that could be construed as a potential conflict of interest.

## Publisher’s Note

All claims expressed in this article are solely those of the authors and do not necessarily represent those of their affiliated organizations, or those of the publisher, the editors and the reviewers. Any product that may be evaluated in this article, or claim that may be made by its manufacturer, is not guaranteed or endorsed by the publisher.
