# Supplementary material for: Increased Cardiovascular Risk in Psoriatic Arthritis: Results From a Case-Control Monocentric Study
Source: Front Med (Lausanne). 2022 May 19;9:785719. doi: 10.3389/fmed.2022.785719 (PMC9160333; doi:10.3389/fmed.2022.785719)
Supplement: Supplementary file 1 [file Data_Sheet_1.docx]

Supplementary Material

Increased cardiovascular risk in psoriatic arthritis: results from a case-control monocentric study

Yannick Degboé, Richard Koch, Laurent Zabraniecki, Bénédicte Jamard, Guillaume Couture, Jean Bernard Ruidavets, Jean Ferrieres, Adeline Ruyssen-Witrand, and Arnaud Constantin

***Supplementary Figure 1: Distribution of cardiovascular risk by deciles with the SCORE and SCORE-PsA equations***

*
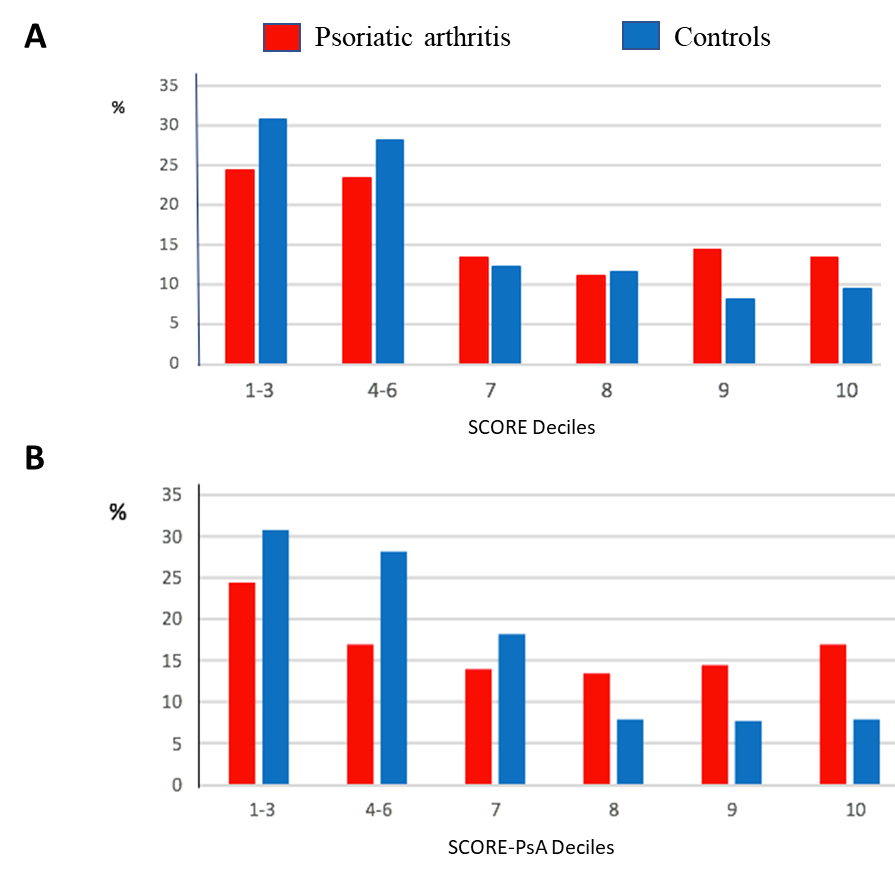
*

***Supplementary Figure 2: Distribution of cardiovascular risk by deciles with the SCORE-PsA equation in 40-65 years old patients***

***
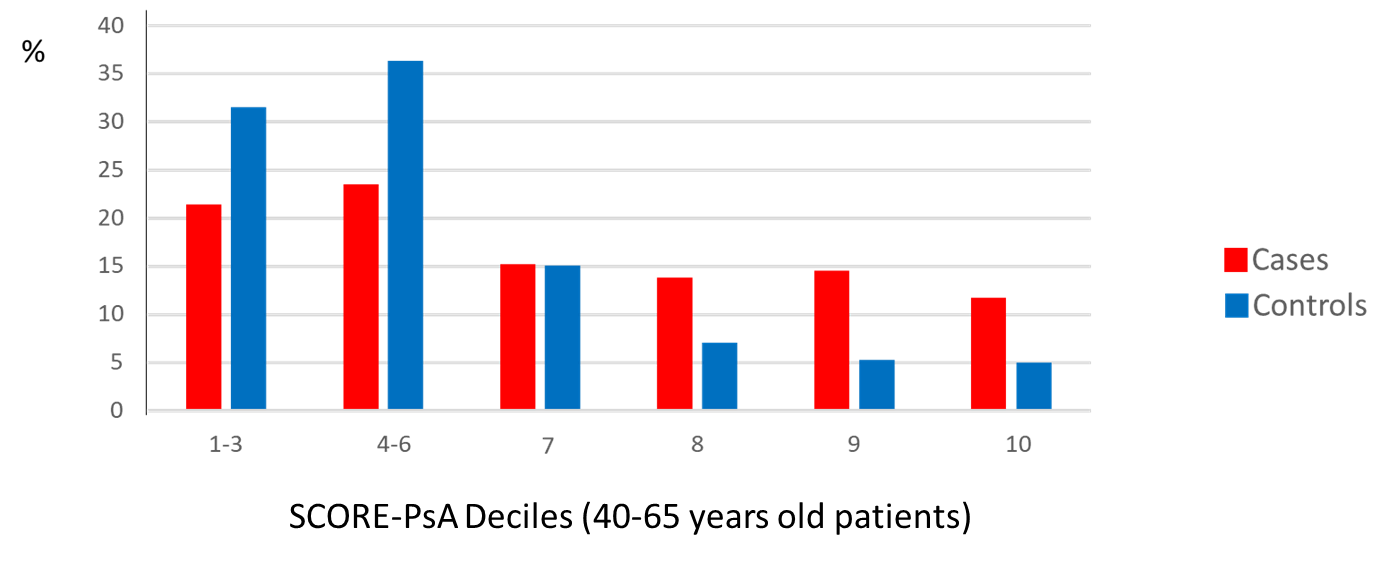
***

***Supplementary Figure 3: Distribution of cardiovascular risk by deciles with QRISK2 and QRISK2-PsA equations***


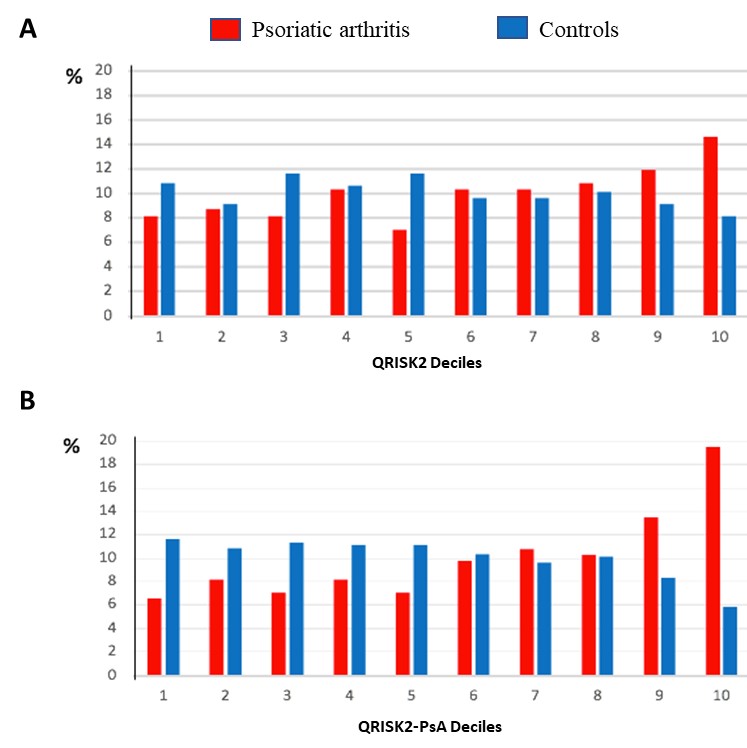


***Supplementary Figure 4: Cardiovascular risk according to QRISK2 and QRISK2-PsA equations***

|  | QRISK2 | QRISK2-PsA |
| --- | --- | --- |
| Number of values | 186 | 186 |
|  |  |  |
| Minimum | 0,2000 | 0,2000 |
| 25% Percentile | 3,100 | 4,200 |
| **Median** | **8,700** | **11,30** |
| 75% Percentile | 17,00 | 21,70 |
| Maximum | 54,70 | 65,10 |
| Range | 54,50 | 64,90 |
|  |  |  |
| **Mean** | **11,24** | **14,31** |
| Std. Deviation | 10,06 | 12,46 |
| Std. Error of Mean | 0,7360 | 0,9108 |

*Comparison of QRISK2 and QRISK2-PsA scores by a Mann-Whitney test.*
